# Aberrant Neural Stem Cell Proliferation and Increased Adult Neurogenesis in Mice Lacking Chromatin Protein HMGB2

**DOI:** 10.1371/journal.pone.0084838

**Published:** 2013-12-31

**Authors:** Ariel B. Abraham, Robert Bronstein, Avanish S. Reddy, Mirjana Maletic-Savatic, Adan Aguirre, Stella E. Tsirka

**Affiliations:** 1 Program in Molecular and Cellular Pharmacology, Stony Brook University, Stony Brook, New York, United States of America; 2 Program in Neuroscience, Stony Brook University, Stony Brook, New York, United States of America; 3 Jan and Dan Duncan Neurological Research Institute at Texas Children’s Hospital, Baylor College of Medicine, Houston, Texas, United States of America; 4 The Medical Scientist Training Program, Stony Brook University, Stony Brook, New York, United States of America; 5 Department of Pharmacological Sciences, Stony Brook University, Stony Brook, New York, United States of America; CSIC/Universidad Autonoma Madrid, Spain

## Abstract

Neural stem and progenitor cells (NSCs/NPCs) are distinct groups of cells found in the mammalian central nervous system (CNS). Previously we determined that members of the High Mobility Group (HMG) B family of chromatin structural proteins modulate NSC proliferation and self-renewal. Among them HMGB2 was found to be dynamically expressed in proliferating and differentiating NSCs, suggesting that it may regulate NSC maintenance. We report now that *Hmgb2^−/−^* mice exhibit SVZ hyperproliferation, increased numbers of SVZ NSCs, and a trend towards aberrant increases in newly born neurons in the olfactory bulb (OB) granule cell layer. Increases in the levels of the transcription factor p21 and the Neural cell adhesion molecule (NCAM), along with down-regulation of the transcription/pluripotency factor Oct4 in the *Hmgb2−/−* SVZ point to a possible pathway for this increased proliferation/differentiation. Our findings suggest that HMGB2 functions as a modulator of neurogenesis in young adult mice through regulation of NSC proliferation, and identify a potential target via which CNS repair could be amplified following trauma or disease-based neuronal degeneration.

## Introduction

Neural stem cells (NSCs) give rise to neural progenitor cells (NPCs) that differentiate during development into all principal cells of the CNS [1], [2]. Postnatally, neurogenesis continues in the subgranular zone (SGZ) of the dentate gyrus (DG) within the hippocampus [3]–[5] and in the subventricular zone (SVZ) [6]–[8]. In the SVZ, NSCs proliferate and give rise to NPCs that migrate via the rostral migratory stream (RMS) to the olfactory bulb (OB) where they terminally differentiate into granule and periglomerular interneurons [6], [8]–[11]. Studies of embryonic and adult NSCs have defined several mitogens that promote NSC proliferation [12]–[16], but the cell autonomous mechanisms controlling NSC self-renewal and maintenance remain unclear. NSC proliferation and neurogenesis in the forebrain decrease with aging in association with increased expression of cyclin-dependent kinase inhibitor p16*^Ink4a^* [17], which is regulated by High mobility group A2 (HMGA2) protein [18]. HMGs are a group of non-histone chromatin proteins that broadly control transcription, replication, recombination, and DNA repair. They bind to nucleosomes and the minor groove of DNA in a sequence-independent manner [19]–[21]. In a previous study, we have identified the HMGB family of chromatin-associated proteins via shotgun proteomics analysis as being differentially expressed in NSCs cultured from the embryonic mouse brain [22]. While HMGB2 is widely expressed during embryogenesis, it becomes restricted primarily to lymphoid organs and the testes postnatally [23]. Mice lacking HMGB2 are described to have defects in spermatogenesis [23] and chondrocyte development [24]. We report here a novel CNS phenotype in which young adult *Hmgb2*-deficient (*Hmgb2−/−*) mice exhibit hyperproliferation in the SVZ niche with aberrantly increased numbers of NSCs, and a trend towards region specific increases in OB neurogenesis. We find that deficiency in *Hmgb2* results in modulation of transcription/pluripotency factors in NSCs such as Oct4, and the eventual downstream modification of Akt signaling. Moreover levels of NCAM 140 are significantly upregulated in the *Hmgb2−/−* SVZ, indicating greater differentiation and migration out of this neural stem cell niche.

## Materials and Methods

All experiments conform to Stony Brook University guidelines on the ethical use of animals and were approved by the Institutional Animal Care and Use Committee. The mice used were C57Bl6 (wild-type, WT), nestin-GFP in the C57Bl6 background, *Hmgb2−/−* in the C57Bl6 background and nestin-GFP-*Hmgb2−/−* in the C57Bl6 background. The *Hmgb2−/−* mouse line was obtained from Drs. Marco Bianchi and Lorenza Ronfani at San Raffaele University. To generate *Hmgb2−/−* mice we cross *Hmgb2* ^+/−^ animals, since the male *Hmgb2−/−* mice are sterile. Littermates are used as controls.

### *In Vivo* Proliferation and Differentiation Assays, Immunofluorescence, and Confocal Imaging

2.5 month old WT and *Hmgb2−/−* mice were given an intraperitoneal injection (IP) of 150 mg/kg BrdU (Sigma) every 12 hours for 2.5 days (5 injections total) and perfused 12 hours after the final injection. Mice were deeply anesthetized using IP injection of 2.5% Avertin solution and transcardially perfused with PBS followed by 4% paraformaldehyde/PBS. The brains were dissected in 4% PFA/PBS at 4°C, and sectioned along the midline. Floating 50 µm thick sagittal sections were generated by vibratome. All brain sections were collected in series. For *in situ* OB characterization, 2-month old WT and *Hmgb2−/−* mice were given an IP injection of 150 mg/kg BrdU (Sigma) every 12 hours for 2 days (4 injections total) and euthanized 14 days after the final injection, at 2.5 months (10 weeks). Perfusion and sectioning were performed as above. For proliferation immunofluorescence (IF) staining, one set of serial sections from each mouse were washed with PBS, and BrdU antigen retrieval was performed using 2N HCL treatment for 1 hour at 37°C followed by two washes in 0.1M Borate Buffer (pH 8) and two washes in 1xPBS. All sections were blocked for 2 hours at room temperature with 10% goat serum/0.3% BSA/0.2% TritonX/PBS solution and stained with rat anti-BrdU antibody (Serotec, 1∶300) and rabbit anti-Ki67 (Abcam, 1∶200) in 0.3% BSA/0.2% TritonX/PBS solution overnight at 4°C. For differentiation IF staining, BrdU antigen retrieval was performed as above. Sections were blocked and then stained with rat anti-BrdU and mouse anti-NeuN (Millipore, 1∶1000) in 0.3% BSA/0.2% TritonX/PBS solution overnight at 4°C. For NSCs/NPCs, serial sections were stained with mouse anti-GFAP (Dako, 1∶500), rabbit anti-DCX (1∶400), and rabbit anti-Mash1 (1∶500) in 0.3% BSA/0.2% TritonX/PBS solution overnight at 4°C. HMGB2 staining was performed as above, with the exclusion of the antigen retrieval step, employing mouse anti-HMGB2 antibody (Abcam ab110193 1: 250). All sections were washed with PBS and stained with highly cross-absorbed secondary antibodies used for dual staining, including anti-rat rhodamine red X (Jackson 1∶500), anti-mouse FITC (1∶200), anti-mouse Cy3 (1∶500), anti-Rabbit Cy5 (1∶500) and anti-Rabbit Alexa 488 (Invitrogen, 1∶2000). All secondary antibody stains were performed in 0.3% BSA/0.2% TritonX/PBS solution at room temperature for 1 hour. Sections were washed extensively with PBS, mounted on Superfrost plus micro slides (VWR), covered with Fluoromount G mounting media (Southern Biotech) and a coverslip (Fisher). A Zeiss LSM 510 confocal system with an Axiovert 200M inverted microscope was used to obtain 100x Z-stack images of the entire thickness of sagittal brain sections containing the anterior SVZ (aSVZ) (proliferation), the proximal rostral migratory stream, and the granule cell and glomerular layers of the OB (differentiation). To determine the number of total proliferating NSCs/NPCs (BrdU+, Ki67+, and BrdU+/Ki67+ cells) in the aSVZ, 10 fields of view comprising the anterior (a)SVZ were imaged using Z-stacks and the cells were quantified using the LSM Image Browser Software (Zeiss). Only proliferating SVZ cells located in the cell dense region around the ventricle were quantified; cells >100 µm from the ependymal layer were excluded to avoid quantifying cells in the striatum. Images of the WT and *Hmgb2−/−* aSVZs were created by layering high magnification fields to construct a composite image. To quantify BrdU+, NeuN+ and BrdU+/NeuN+ newborn neurons in the OB GCL and GL, Z-stacks were generated from 3 random fields in each OB layer and analyzed using LSM software. To quantify nestin-GFP+/GFAP+ cells we marked the fluorescence in the nestin-GFP transgenic mice within the cell body of the nestin+ neural progenitors. We then stained for GFAP using a second fluorophore (red or far red), thus making it possible to visualize GFAP processes coming off the cell bodies of GFP+ neural progenitors, whose cell body was clearly green. This was further determined following use of Z-stacks and 3D reconstructions of Z-stack data on the confocal microscope within the neurogenic niches. NestinGFP+ processes also stained positive for GFAP allowing to see co-localization of the processes extending from the NestinGFP+ cell bodies.

### SVZ Wholemount Dissection

Tissue comprising the lateral ventricle wall (SVZ) was dissected from 10-week old *Nestin-GFP+ Hmgb2* WT and KO mice according to established methods [25].

### SVZ Adherent Monolayer Culture

Tissue comprising the lateral ventricle wall was dissected from 10-week old *Nestin-GFP+ Hmgb2* WT and KO mice in cold PBS containing 2% glucose (NeuroCult). Following dissection the tissue was mechanically and enzymatically (NeuroCult Enzymatic Dissociation kit) dissociated and plated in 10 cm coated (Poly-D-Lysine 100 µg/ml, Laminin 10 µg/ml) tissue culture plates. Complete NeuroCult proliferation media were supplemented with recombinant human epidermal growth factor (rhEGF, 20 ng/ml) and recombinant human fibroblast growth factor (rhFGF, 10 ng/ml) as well as 0.2% Heparin.

### RT-PCR

Total RNA was isolated out of dissected SVZ wholemount tissue from 10 week old *Nestin-GFP*+ *Hmgb2* WT and KO mice according to the RNA Bee (AMSBIO) protocol. cDNA was reverse transcribed using the High Capacity cDNA Reverse Transcription Kit (Applied Biosystems). The PCR runs were completed on a Veriti 96-well thermacycler (Applied Biosystems). The primers for the HMGB2 RT-PCR run were as follows:

HMGB2 Forward Primer:

TGTCCTCGTACGCCTTCTTC

HMGB2 Reverse Primer:

CCTCCTCATCTTCTGGTTCG

### Western Blots

Two sample populations were employed in the western blot analysis, whole cell lysates of NSC monolayer cultures from the SVZ as well as whole cell lysates of dissected SVZ wholemount tissue. Protein concentrations were determined via DC Assay (Biorad). Proteins were loaded into 10 and 12% Tris Glycine SDS-PAGE, transferred to PVDF, blocked with 5% BSA/TBS-T (pAkt) or 5% Milk/TBS-T, and incubated overnight at 4° with primary antibodies: rabbit anti-Oct4 (Abcam, 1∶300), rabbit anti-Akt (Cell Signaling, 1∶1000), rabbit anti-PhosphoAkt Ser473 (Cell Signaling, 1∶2000), mouse anti-p21 (BD Pharmingen, 1∶1000), rabbit anti-NCAM (Cell Signaling, 1∶1000). Membranes were washed 3x with TBS-T and incubated for 1 hr at room temperature with either Goat anti-Rabbit HRP conjugated secondary antibody (Invitrogen, 1∶2500), or Rabbit anti-Mouse HRP conjugated secondary antibody (Invitrogen, 1∶2500) followed by 3x washes with TBS-T, a one minute incubation in Pierce ECL western blotting substrate and exposure in the dark room.

### Statistics

All comparisons were conducted using either Kruskal-Wallis one-way analysis of variance by ranks or Wilcoxon signed-rank test. Statistical significance cut off for all comparisons was p≤0.05.

## Results

### HMGB2 is Present in the Postnatal SVZ at the Transcript and Protein Level

HMGB2 mRNA was previously reported to be present in the SVZ of P90 adult mice [26]. We verified expression of HMGB2 protein in SVZ progenitor cells in brain sections of young adult Nestin-GFP^+^ transgenic mice (Fig. S1A–C). HMGB2 was primarily expressed in the Nestin-GFP^+^ progenitors (arrowheads) although not all of the Nestin+ SVZ cells expressed HMGB2 (Fig. S1D). RT-PCR analysis of SVZ wholemount tissue from *Hmgb2+/+* and *Hmgb2−/−* mice also demonstrated the ample presence of HMGB2 transcripts (Fig. S1E). We also investigated whether HMGB2 could be detected in a very homogenous NSC population: NSC (Fig. S1G) cultures from SVZ were plated and did show high levels of HMGB2 protein in the *Hmgb2+/+* SVZ (Fig. S1F).

### Hyperproliferation and Altered NSC/NPC Composition in the SVZ of Young Adult *Hmgb2−/−* mice

**Figure 1:**
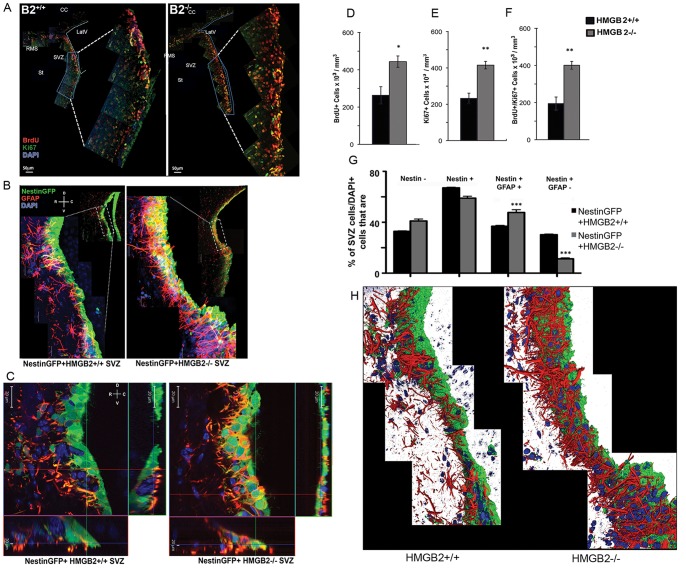
Hyperproliferation and altered NSC/NPC composition *in vivo* in 10 week old *Hmgb2−/−* mice. A) Immunostaining for BrdU (red) and Ki67 (green) in the anterior SVZ of 10 week old WT and *Hmgb2−/−* mice. B) Collapsed image of confocal Z-stacks from brain sections of compound *Hmgb2+/+*NestinGFP+ and *Hmgb2−/−*NestinGFP+ transgenic mice stained for GFAP, a marker of SVZ NSCs. GFAP (red), Nestin (green), DAPI (blue). (C) High magnification orthogonal view of a single confocal plane from brain sections of compound transgenic mice stained with GFAP at the SVZ/RMS junction. D) Quantification of SVZ BrdU+ cells and cell density in (A), E) Ki67+ cells and cell density in (A), F) BrdU+/Ki67+ cells and cell density in (A). All figures are mean+/− SEM, *p≤0.05, **p≤0.005. n = 4 WT and n = 5 *Hmgb2−/−* per group. (G) Quantification of Nestin+ and GFAP+ staining in WT and *Hmgb2−/−* SVZ in (B). All figures are mean+/− SEM; the Kruskal-Wallis test was used. ***p = 0.0002. n = 4 WT and n = 5 *Hmgb2−/−* per group. (H) Reconstruction of z-stacks used to generate the image in panel B of GFAP staining in the SVZ of *Hmgb2+/+* (WT) and *Hmgb2−/−* mice. DAPI (blue), GFAP (red), and Nestin (green).

To assess a potential effect of HMGB2 deficiency on proliferation in the SVZ stem cell niche, we injected 10-week old mice with BrdU (150 µg/mg) every 12 hours for 2.5 days (5 injections total) and immunostained brain sections for BrdU incorporation as an S phase marker and the Ki67 as a pan cell-cycle proliferation marker. In the anterior SVZ (aSVZ), the BrdU+, Ki67+ and BrdU+/Ki67+ cell numbers and densities were elevated in *Hmgb2−/−* mice relative to WT animals (Fig. 1A,D–F). *Hmgb2−/−* mice reached a mean cell density of 4×10^5^ BrdU+/Ki67+ cells per mm^3^ in the aSVZ, twice the density observed in the WT mice [2×10^5^ BrdU+/Ki67+ cells per mm^3^ (Mean+/−SEM, n = 4 WT and n = 5, p<0.005)]. To further characterize the hyperproliferating cells in the SVZ of 10-week *Hmgb2−/−* mice, we crossed *Hmgb2*+/− mice [23] with Nestin-GFP+ transgenic mice [27]. The distribution of SVZ NSCs was examined using the marker GFAP [6], [28]–[31]. In Nestin*Gfp+Hmgb2+/+* mice, GFAP expression was present in the SVZ and co-localized with NestinGFP+ processes of NSCs/NPCs (Fig. 1B). In Nestin*Gfp+Hmgb2−/−* mice, there was a dramatic increase in GFAP+ processes in the SVZ that appeared to arise from NestinGFP+ cell bodies (Fig. 1B), suggesting increased numbers of Nestin+GFAP+ NSCs in the SVZ. An analysis of SVZ cell composition using orthogonal views confirmed increased GFAP expression in Nestin+ NSCs in *Hmgb2−/−* mice compared to WT mice (Fig. 1C,G, Fig. S2), and 3-D reconstruction of WT and *Hmgb2−/−* SVZ confirmed this increase in NSCs (Fig. 1H). Quantification of NestinGFP+ cells, subdivided into NestinGFP+GFAP+ and NestinGFP+GFAP- cell populations, suggests that *Hmgb2−/−* mice have a higher percentage of NestinGFP+GFAP+ and lower percentages of NestinGFP+GFAP- cells than age-matched WT mice (Fig. 1G).

### Decrease in Mash1+ Transient Amplifying Cells (TACs) and Increase in Doublecortin+ Neuroblasts in the SVZ of *Hmgb2−/−* Mice

**Figure 2:**
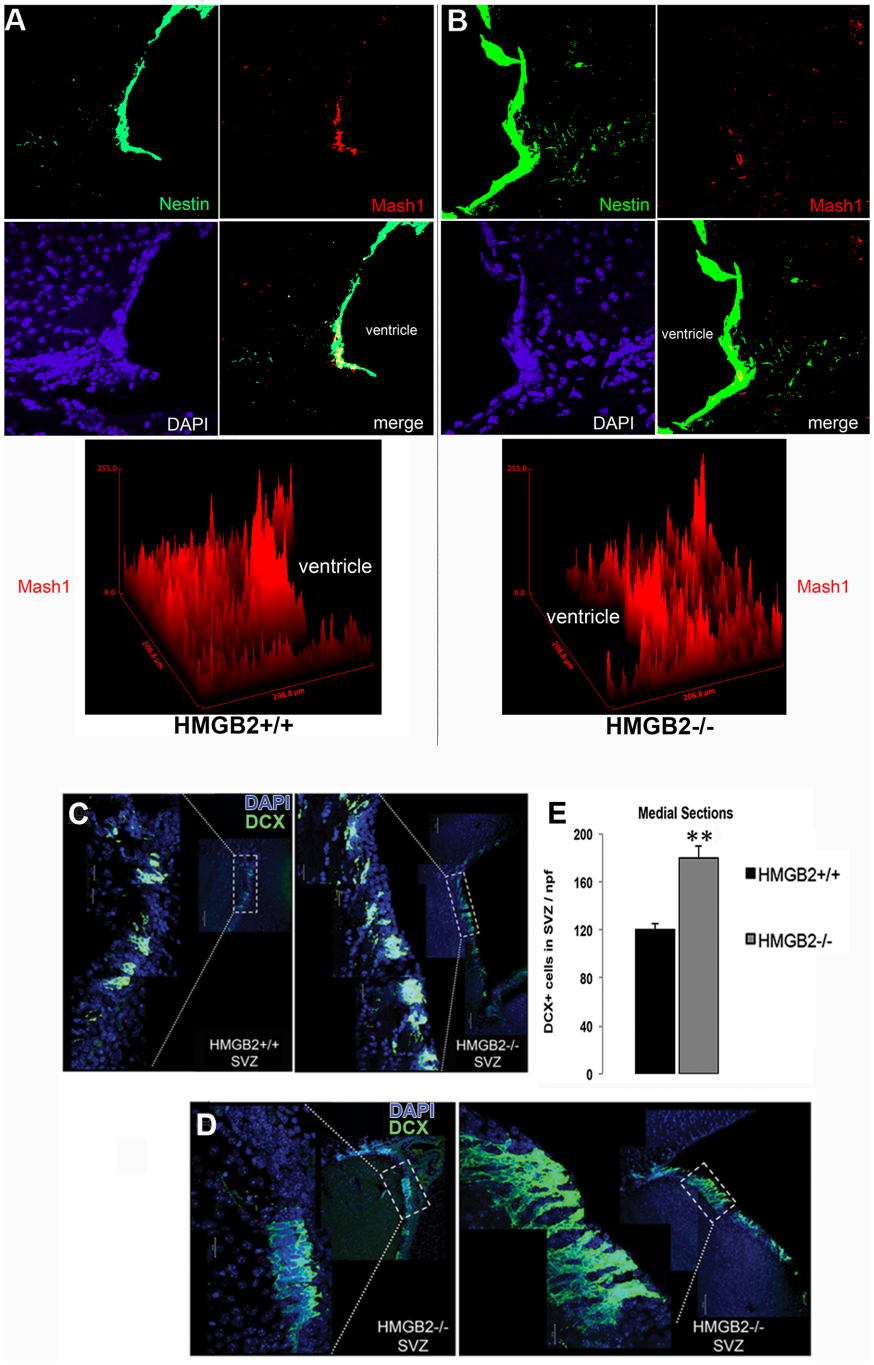
Heightened DCX expression in the SVZ of 10 week old *Hmgb2−/−* mice. Mash1 and Nestin expression in lateral *Hmgb2+/+* and *Hmgb2−/−* brain sections and surface plots of pixel density distributions (A,B). Doublecortin (DCX) in lateral and medial *Hmgb2+/+* and *Hmgb2−/−* brain sections (C,D) with quantification of DCX+ cells/SVZ hpf in WT and *Hmgb2−/−* medial brain sections (E). The values are mean+/− SEM, **p = 0.036.

We further assessed the NPC population by examining expression of doublecortin (DCX), a marker of neuroblasts (cells committed to the neuronal lineage), as well as Mash1, which is a transcription factor expressed by TACs in the SVZ and by intermediate progenitors (IP) in the SGZ [32]. In lateral sagittal sections, Mash1+ TACs and DCX+ neuroblasts clustered in WT mice in the SVZ and outlet to the rostral migratory stream (RMS) (Fig. 2A,C). In *Hmgb2−/−* mice the density of Mash1 staining was dramatically decreased closer to the ventricle (Fig. 2B). The surface plot (Fig. 2B) depicts the drop in TACs in close proximity to the ventricular wall in the *Hmgb2−/−* SVZ. In contrast, the clusters of DCX+ NPCs were larger, and most prominent in more medial sagittal sections (Fig. 2D). The densely-packed DCX+ cords within the *Hmgb2−/−* SVZ contained almost 50% more cells per high-powered field than age matched WT mice (Fig. 2E).

### Increased Neurogenesis in the Olfactory Bulb Granule Cell Layer, but not Glomerular Layer Neurogenesis in *Hmgb2−/−* Mice

**Figure 3:**
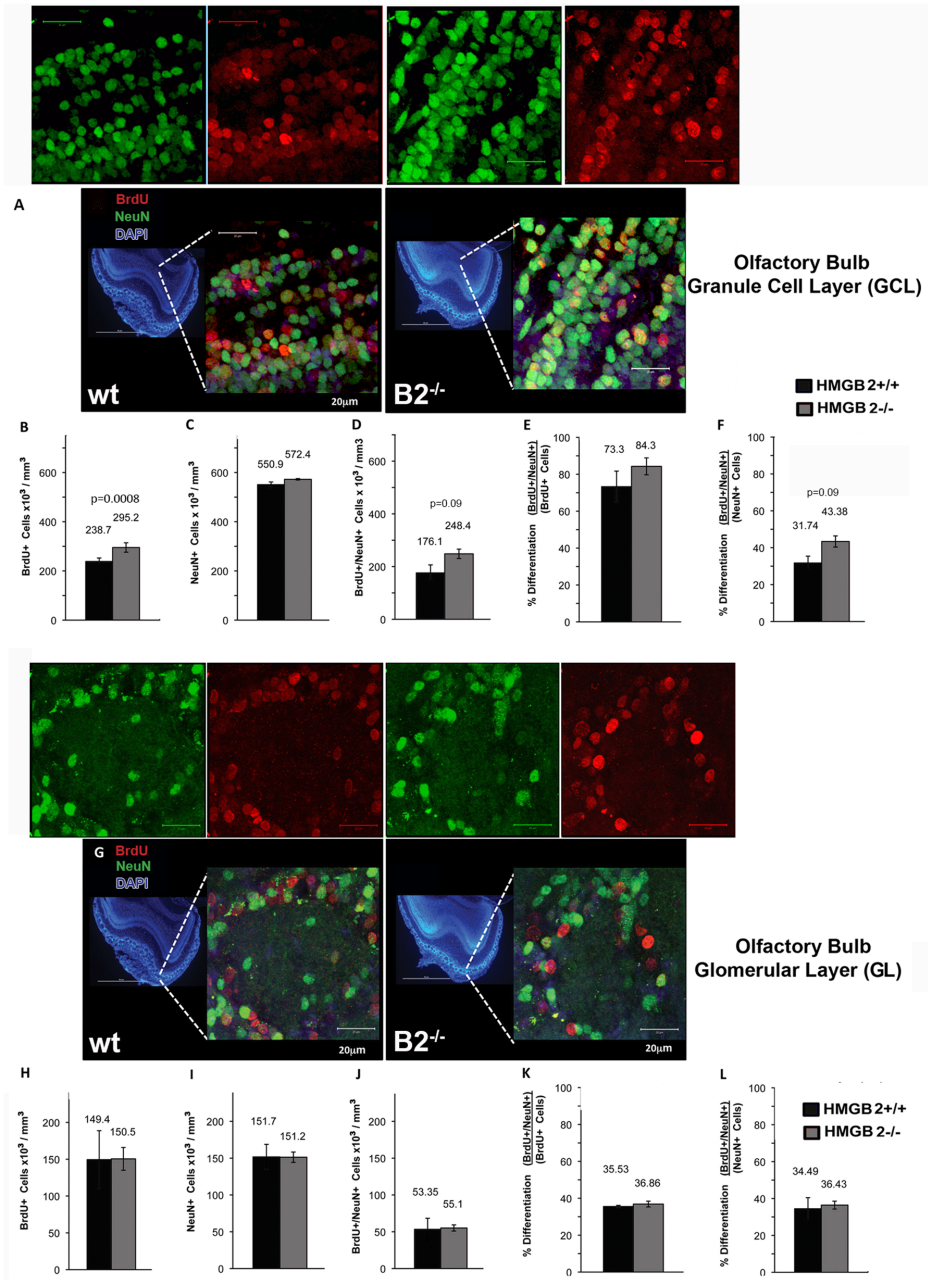
Increase in olfactory bulb granule cell layer neurogenesis in 10 week old *Hmgb2−/−* mice. A) Immunostaining for BrdU (red) and NeuN (green) and the merged image in the OB granule cell layer (GCL) of 10 week old mice after 14 days of differentiation. Quantification of (B) BrdU+, (C) NeuN+, and (D) BrdU+/NeuN+ cell densities in the GCL of WT and *Hmgb2−/−*10 week old mice. Quantification of the relative fraction of newborn GCL neurons (BrdU+/NeuN+) among (E) all BrdU+ cells and (F) all NeuN+ cells. (G) Immunostaining of OB glomerular layer (GL) neurogenesis after 14 days differentiation *in vivo*. (H-L) Quantification of GL neurogenesis at 2.5 months. All figures are mean+/− SEM, n = 2 WT and n = 3 *Hmgb2−/−* per group; the Kruskal-Wallis test was used. (p = 0.0008 for panel B), but no significance for all other panels.

Given the changes in NSCs, NPCs, and DCX+ neuroblasts in *Hmgb2−/−* mice, we explored the differentiation of HMGB2 SVZ cells in the olfactory bulb. BrdU (150 mg/kg) was injected every 12 hours for 2 days in 8-week old WT and *Hmgb2−/−* mice. Tissues were recovered at 10-weeks when the labeled NSCs/NPCs cells had migrated to the OB and differentiated into neurons in the granule cell layer (GCL) and the glomerular layer (GL). There was a 25% increase in BrdU+ cell numbers in the GCL of *Hmgb2−/−* mice compared to WT mice (Fig. 3A,B), but no difference in the total neuronal (NeuN) cell numbers in the GCL (Fig. 3C). The total BrdU+/NeuN+ in the GCL of the *Hmgb2−/−* mice exhibited a trend towards higher numbers relative to WT mice (Fig. 3D). The percentage of newborn neurons (84.3%) among all BrdU+ cells was elevated in the GCL of mice relative to WT mice (73.3%) (Fig. 3E), suggesting possible accelerated differentiation/maturation of newborn GCL neurons in the *Hmgb2−/−* mice. The percentage of newborn neurons present among all GCL neurons in the *Hmgb2−/−* mouse was 43.38% (Fig. 3F), and constituted 31.74% of all neurons in 10-week WT mice, suggesting that the proportion of newborn GCL neurons in the *Hmgb2−/−* mouse may be greater than the proportion of newborn GCL neurons in WT mice of the same age. We did not detect changes in GL neurogenesis in *Hmgb2−/−* mice. BrdU+ cells, NeuN+ cells or BrdU+/NeuN+ cells in the GL of the *Hmgb2−/−* OB at 10 weeks of age remained largely unchanged compared to age matched WT mice (Fig. 3G–L). Additionally, the relative number of newborn GL neurons, as a percentage of BrdU+ or percentage of NeuN+ cells (Fig. 3K,L), did not significantly differ between WT and *Hmgb2−/−* mice, suggesting that GL neurogenesis is normal in the *Hmgb2−/−* mice at this age.

### Pluripotency and Neuroblast Differentiation Factors are Variably Expressed in *Hmgb2*−/− Mouse SVZ

**Figure 4:**
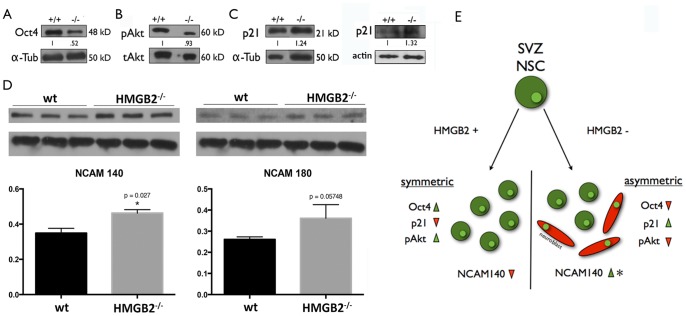
Pluripotency and migration/differentiation factors are variably expressed in the *Hmgb2−/−* SVZ neural stem cell niche. The protein levels of different factors were examined in SVZ wholemount tissue lysates from wt and *Hmgb2* ***−/−*** mice. A) Oct4; B) pAkt (ser); C) p21 (two representative immunoblots are provided); D) NCAM (n = 3); E) Cartoon depicting the suggested role of HMGB2 in NSC maintenance.

A recent report points to a strong interaction between HMGB2 and the pluripotency factor Oct4 in embryonic stem (ES) cells [33]. We examined the Oct4 levels in vivo, by isolating protein lysates from wholemount dissections of SVZ tissue, which contains all the cells comprising the neurogenic niche along the lateral ventricle. A dramatic reduction (∼50%) in Oct4 levels was observed in the *Hmgb2−/−* SVZ wholemount tissue lysates (Fig. 4A), accompanied by reduced pAkt (Fig. 4B). Our data also revealed the upregulation of the tumor suppressor protein p21 in the *Hmgb2−/−* SVZ lysates (Fig. 4C). We also tested the levels of various isoforms of the neural cell adhesion molecule (NCAM) in the SVZ tissue lysates, and found a significant increase in NCAM140 expression [34] indicating more robust differentiation/migration in the *Hmgb2−/−* SVZ (Fig. 4D). Taken together, these data demonstrate that HMGB2 loss depletes pluripotency factors from the SVZ while increasing molecules such as NCAM, which potentiate differentiation and migration out of the niche.

## Discussion

In this report we unveil a role of HMGB2 in adult SVZ neurogenesis. Using wild-type and *Hmgb2−/−* mice we observed a significant increase in BrdU incorporation in the NestinGFP+/GFAP+ NSC population in the *Hmgb2−/−* animals. The number of Nestin+GFAP+ proliferating NSCs exceeded the number of Nestin+GFAP- proliferating NPCs. As a correlate for the Nestin+GFAP- population, we stained for TAC marker Mash1 and saw a reduction in these cells within the *Hmgb2−/−* SVZ. The Mash1+ cells were also Nestin+, indicating that they are in fact adult TACs. We also observed a nearly 50% increase in neuroblast lineage commitment in the *Hmgb2−/−* SVZ niche, which could suggest a quick depletion of the TAC population and increased neurogenesis. We investigated the downstream outcomes of this increased neuroblast production on the OB, which is the primary output site for adult murine SVZ neurogenesis. Although no gross changes in OB glomerular layer newborn interneuron population were obvious, there was a trend towards increase in interneuron numbers in the OB granule cell layer, possibly reflecting region-specific increases in SVZ neurogenesis in the *Hmgb2−/−* [35].

We find that a high proportion of NeuN neurons are new (Fig. 3F). Although this number is higher than others previously reported in the literature, there are several explanations for our finding:

The BrdU injection was not done once but rather multiple times over the course of several days. Previous work [36] has shown that multiple injections of BrdU increase the probability of labeling a stem cell with BrdU in the SGZ hippocampal stem cell niche. This is because the NSCs are quiescent and cycle fewer times than amplifying neural progenitor cells. Therefore this repeat labeling approach labels many cells with BrdU, including in the SVZ, RMS, and even in the OB.
The age of the mice and the fact that the mice were euthanized on day 14 of differentiation result in euthanization prior to periods of “pruning back” cell numbers due to prior to activity-dependent survival [37]. In the same paper Patreanu and Alvarez-Buylla argue that the description of 14% ‘new’ cells in the GCL ‘is probably an underestimation because it assumes that with each daily injection all of the neurons born that day were labeled’.
We used an antigen retrieval step to allow for better staining by the rat anti-BrdU antibody. This may (or may not have) increased the NeuN antibody’s ability to bind its epitope.
The NeuN antibody used a clean antibody and was titrated down to minimize non-specific staining, and used different species for the secondary Abs, so there is likely no cross reactivity to drive up the numbers artificially.

Our previous work using shotgun proteomics analysis revealed changes in HMGB family expression in NSCs [22]. The embryonic B2 expression pattern resembles that of NSC HMGA2 [18]. It is higher in the NSC proliferative compartment of the embryonic telencephalon (ventricle zone) than in the differentiated compartment (cortex). Support of this result in another system can be seen in mesenchymal stem cells (MSCs), where the HMGB2 is highly expressed in undifferentiated MSCs and is significantly downregulated during chondrogenesis and osteogenesis [38]. A distinct mechanism for HMGB2 action in the nucleus of MSCs implicates its binding to Lef1, which is a key player in the β-catenin transcriptional program [38].

It has been suggested that HMGB2 binding to the transcription factor Oct4 is important for maintaining the pluripotent state of embryonic stem cells (ES) [33]. Oct4 levels were greatly decreased in whole cell lysate from *Hmgb2−/−* SVZ tissue. The tissue is considerably heterogeneous representing NSC/NPC populations and differentiating cells such as neuroblasts. The changes in Oct4 levels in the Hmgb^−/−^ SVZ were presumably due to increased NPC differentiation into the neuroblast lineage. Decreases in Oct4 levels are accompanied by reduction in pAkt(Ser) in ES cells as seen in the *Hmgb2−/−* SVZ [33]. Increases in p21 levels, a tumor suppressor protein, have also been reported when Oct4 is downregulated, as observed in our SVZ tissue samples [39]. Supporting the assertion that reduction in pluripotency factors in concomitant with increased differentiation and early exit of neuroblasts from the SVZ niche we tested levels of NCAM in SVZ lysates and found a significant reduction of the NCAM 140 isoform, a molecule known to be upregulated during increased migration/differentiation of SVZ progenitors [34]. The results presented here strongly suggest a role for HMGB2 in NSC proliferation/differentiation dynamics pointing to a novel HMGB2-dependent stem cell phenotype in the CNS.

## Supporting Information

Figure S1 **Immunostaining, RT-PCR and immunoblots of HMGB2 in NestinGFP+ (WT) transgenic mice.** A) DAPI staining of SVZ in NestinGFP+*Hmgb2+/+* mouse. B) HMGB2 staining of SVZ in NestinGFP+*Hmgb2+/+* mouse. C) Nestin expression in NestinGFP+*Hmgb2+/+* mouse. D) A–C superimposed image. E) HMGB2 transcript levels in SVZ wholemount tissue total RNA samples. F.) HMGB2 protein levels in NSC monolayer cultures from wt and *Hmgb2−/−* mice. G) An example of an NSC in stem cell monolayer culture from the nestinGFP mouse SVZ.
(TIF)

Figure S2 **Collapsed image of confocal Z-stacks from brain sections of compound** ***Hmgb2+/+NestinGFP+*** **and** ***Hmgb2−/−NestinGFP+*** **transgenic mice.** In the sections through the SVZ Nestin-GFP (green) expression and GFAP (red) levels are shown. These sections are the same as in Fig. 1B, but the red and green channels are separated.
(TIF)
