# Supplementary figures and images for: Aberrant Neural Stem Cell Proliferation and Increased Adult Neurogenesis in Mice Lacking Chromatin Protein HMGB2
Source: PLoS One. 2013 Dec 31;8(12):e84838. doi: 10.1371/journal.pone.0084838 (PMC3877347; doi:10.1371/journal.pone.0084838)

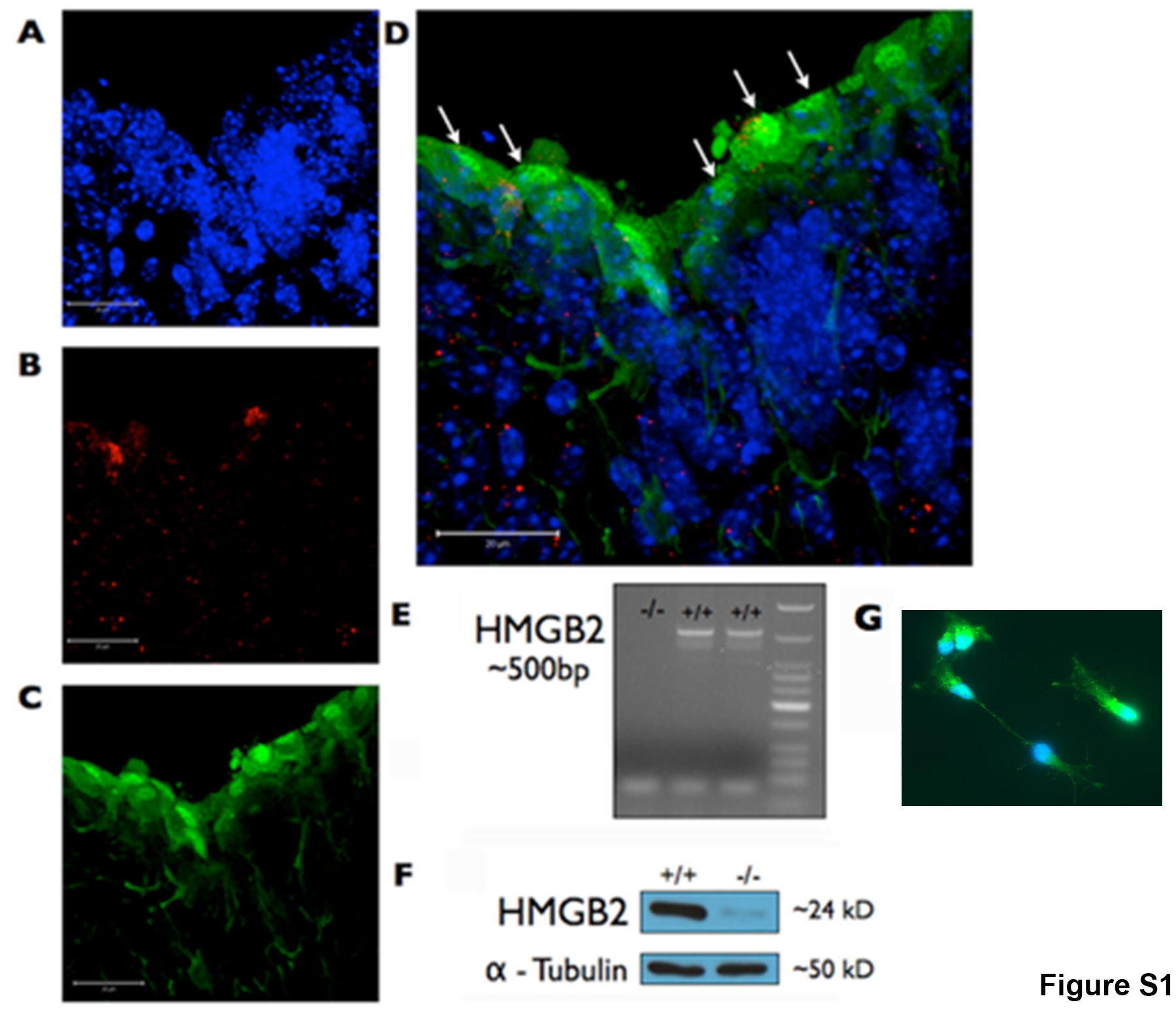

Supplement: Figure S1 — Immunostaining, RT-PCR and immunoblots of HMGB2 in NestinGFP+ (WT) transgenic mice. A) DAPI staining of SVZ in NestinGFP+Hmgb2+/+ mouse. B) HMGB2 staining of SVZ in NestinGFP+Hmgb2+/+ mouse. C) Nestin expression in NestinGFP+Hmgb2+/+ mouse. D) A–C superimposed image. E) HMGB2 transcript levels in SVZ wholemount tissue total RNA samples. F.) HMGB2 protein levels in NSC monolayer cultures from wt and Hmgb2−/− mice. G) An example of an NSC in stem cell monolayer culture from the nestinGFP mouse SVZ. (TIF) [file pone.0084838.s001.tif]

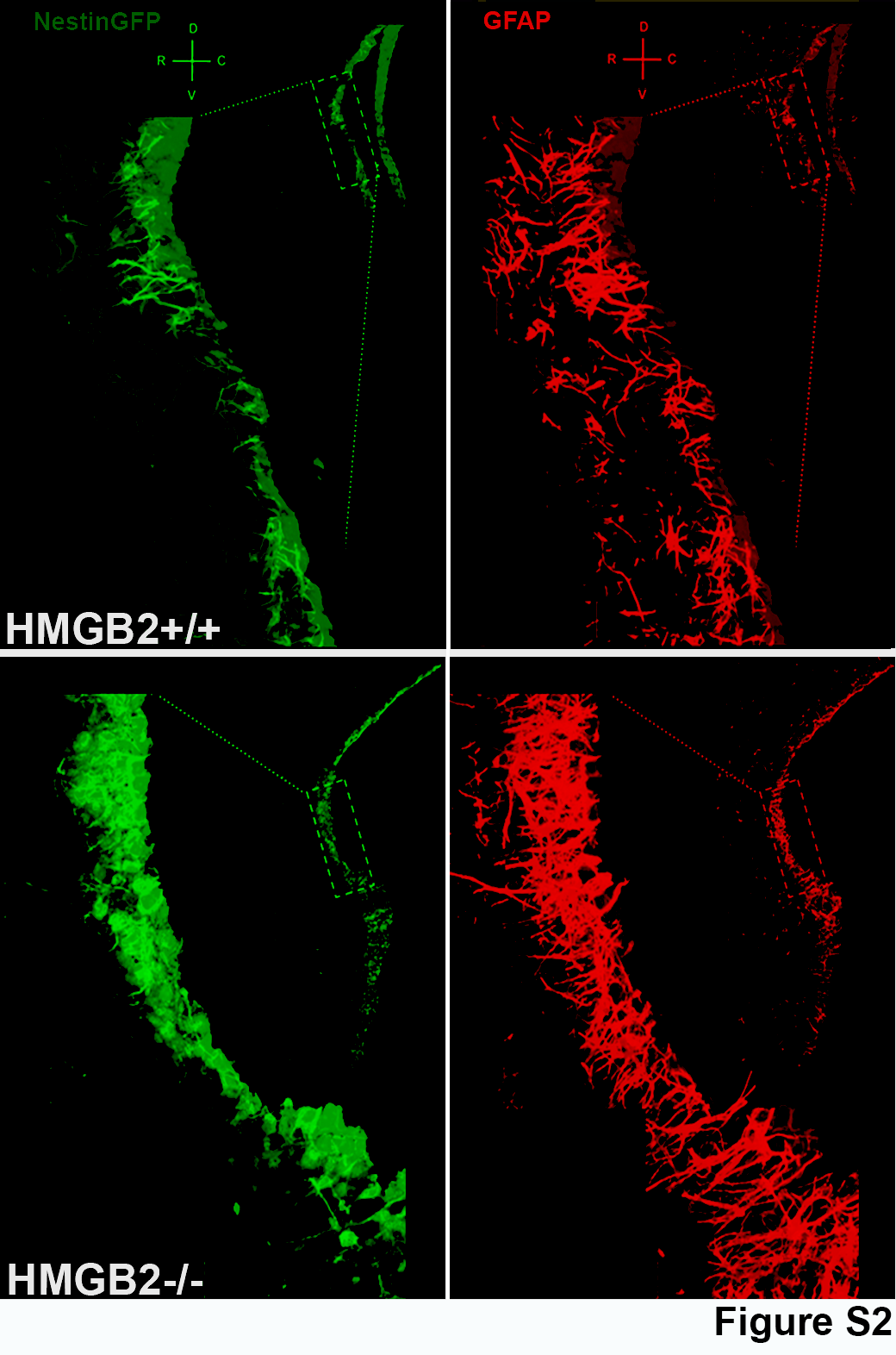

Supplement: Figure S2 — Collapsed image of confocal Z-stacks from brain sections of compound Hmgb2+/+NestinGFP+ and Hmgb2−/−NestinGFP+ transgenic mice. In the sections through the SVZ Nestin-GFP (green) expression and GFAP (red) levels are shown. These sections are the same as in Fig. 1B, but the red and green channels are separated. (TIF) [file pone.0084838.s002.tif]
